# Predictive role of atrial fibrillation in cognitive decline: a systematic review and meta-analysis of 2.8 million individuals^[Author-notes euac003-FM2]^

**DOI:** 10.1093/europace/euac003

**Published:** 2022-01-21

**Authors:** Yu Han Koh, Leslie Z W Lew, Kyle B Franke, Adrian D Elliott, Dennis H Lau, Anand Thiyagarajah, Dominik Linz, Margaret Arstall, Phillip J Tully, Bernhard T Baune, Dian A Munawar, Rajiv Mahajan

**Affiliations:** The University of Adelaide, Adelaide, Australia; The University of Adelaide, Adelaide, Australia; The University of Adelaide, Adelaide, Australia; The University of Adelaide, Adelaide, Australia; The University of Adelaide, Adelaide, Australia; Royal Adelaide Hospital, Adelaide, Australia; Royal Adelaide Hospital, Adelaide, Australia; The University of Adelaide, Adelaide, Australia; The University of Adelaide, Adelaide, Australia; Lyell McEwin Hospital, Adelaide, Australia; The University of Adelaide, Adelaide, Australia; Department of Psychiatry, University of Melbourne, Melbourne, Australia; The Florey Institute of Neuroscience and Mental Health, The University of Melbourne, Parkville, Victoria, Australia; Department of Psychiatry, University of Münster, Münster, Germany; The University of Adelaide, Adelaide, Australia; Royal Adelaide Hospital, Adelaide, Australia; Department of Cardiology and Vascular Medicine, University of Indonesia, Jakarta, Indonesia; The University of Adelaide, Adelaide, Australia; Lyell McEwin Hospital, Adelaide, Australia

**Keywords:** Atrial fibrillation, Cognitive impairment, Dementia, Meta-analysis, Silent brain infarction, Stroke, Cerebral small vessel disease

## Abstract

**Aims:**

To systematic review and meta-analyse the association and mechanistic links between atrial fibrillation (AF) and cognitive impairment.

**Methods and results:**

PubMed, EMBASE, and Cochrane Library were searched up to 27 March 2021 and yielded 4534 citations. After exclusions, 61 were analysed; 15 and 6 studies reported on the association of AF and cognitive impairment in the general population and post-stroke cohorts, respectively. Thirty-six studies reported on the neuro-pathological changes in patients with AF; of those, 13 reported on silent cerebral infarction (SCI) and 11 reported on cerebral microbleeds (CMB). Atrial fibrillation was associated with 39% increased risk of cognitive impairment in the general population [*n* = 15: 2 822 974 patients; hazard ratio = 1.39; 95% confidence interval (CI) 1.25–1.53, *I*^2^ = 90.3%; follow-up 3.8–25 years]. In the post-stroke cohort, AF was associated with a 2.70-fold increased risk of cognitive impairment [adjusted odds ratio (OR) 2.70; 95% CI 1.66–3.74, *I*^2^ = 0.0%; follow-up 0.25–3.78 years]. Atrial fibrillation was associated with cerebral small vessel disease, such as white matter hyperintensities and CMB (*n* = 8: 3698 patients; OR = 1.38; 95% CI 1.11–1.73, *I*^2^ = 0.0%), SCI (*n* = 13: 6188 patients; OR = 2.11; 95% CI 1.58–2.64, *I*^2^ = 0%), and decreased cerebral perfusion and cerebral volume even in the absence of clinical stroke.

**Conclusion:**

Atrial fibrillation is associated with increased risk of cognitive impairment. The association with cerebral small vessel disease and cerebral atrophy secondary to cardioembolism and cerebral hypoperfusion may suggest a plausible link in the absence of clinical stroke. PROSPERO CRD42018109185.

## Graphical Abstract

**Graphical Abstract:**
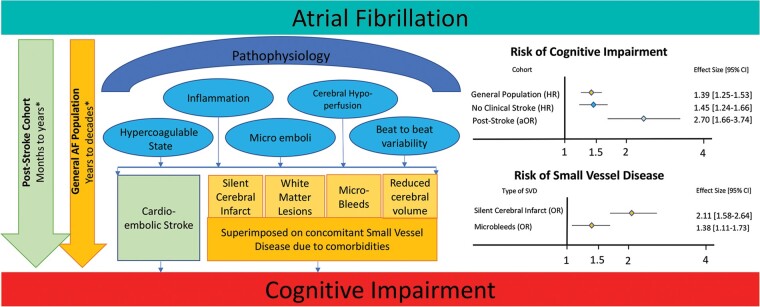

**What’s new?**

- Atrial fibrillation (AF) is associated with a 39% increased risk of cognitive impairment.
- The cognitive impairment was early and more frequent after clinical stroke in presence of AF.
- Cerebral small vessel disease, such as white matter hyperintensities, microbleeds, silent cortical and subcortical infarction, and decrease in cerebral volume, represents the plausible link between AF and cognitive impairment.

## Introduction

Atrial fibrillation (AF) and cognitive impairment are important public health problems and represent significant burden on health resources.^1,^^2^ Population studies have suggested an association between AF and cognitive impairment. Atrial fibrillation and cognitive impairment share similar risk factors, such as age, diabetes, hypertension, and heart failure, which could confound the association.^3–5^ In addition, stroke, a serious complication of AF, is a well-described risk factor for cognitive impairment.^6^ Silent cerebral emboli and chronic hypoperfusion during AF may also represent a plausible pathophysiological link between AF and cognitive impairment, though evidence is unclear.

Cognitive impairment is defined as a decline from a previous level of performance in one or more cognitive domains (complex attention, executive function, learning and memory, language, perceptual motor, or social cognition). It is designated ‘mild’ when it does not interfere with the capacity for independence. On the other hand, severe cognitive impairment (dementia) is a more severe form that interferes with daily function, usually defined either by the Diagnostic and Statistical Manual of Mental Disorders (DSM) criteria or International Classification of Diseases (ICD) codes.

In this study, we undertook a meta-analysis and systematic review of (i) studies evaluating the association between AF and cognitive impairment, and (ii) neuropathological lesions in AF patients to better characterize the mechanistic link between AF patients and cognitive impairment.

## Methods

This systematic review complies with the consensus statement outlined by the Meta-analysis of Observational Studies in the Epidemiology group and *P*referred *R*eporting *I*tems for *S*ystematic *R*eview and *M*eta-*A*nalysis statements.^7^ The meta-analysis was registered with the PROSPERO International prospective register of systematic reviews (CRD42018109185).

### Search strategy

The English scientific literature was searched using PubMed, EMBASE, and Cochrane Library from their inception to 27 March 2021 with the assistance of an experienced librarian. The following keywords: atrial fibrillation, cognitive impairment, major neurocognitive disorder, microbleeds, silent cerebral infarcts, and dementia were used (Supplementary material online, *Methods S1*).

### Inclusion and exclusion criteria

Only prospective studies were included for the meta-analysis evaluating the relationship between AF and cognitive impairment. Furthermore, studies reporting neuropathological lesions in the AF population were included and reported in a systematic manner. The exclusion criteria were (i) reviews, editorials, letters, case series, case reports, and conference proceedings; (ii) sample size <50; (iii) studies lacking a control group; (iv) studies in which the control group was selected from patients with other types of arrhythmias; (v) studies where the measure of association was not reported; and (vi) studies that provided unadjusted analyses. Where multiple studies described the same population (sub- and follow-up studies), the study with the most comprehensive data was included.

### Study selection and data extraction

The study selection and data extraction were performed using the predefined inclusion and exclusion criteria. Although review articles were excluded, their reference lists were examined for potentially relevant publications. During data extraction, information was collected on the study design, length of follow-up, outcome measures, method of assessing outcomes, inclusion/exclusion criteria, and results. The maximally adjusted risk ratios were extracted and pooled across studies. The data were reviewed by two authors independently (Y.H.K. and L.L.) and disagreements were resolved by consensus with the help of a third investigator (R.M.). The methodological qualities of the included studies were assessed using the modified Newcastle-Ottawa Scale. The outcomes of the meta-analysis were defined as (i) association of AF and cognitive impairment and (ii) impact of AF on progression of cognitive impairment. The outcome of the systematic review was to systematically define the neuro-pathological changes in AF.

### Statistical analysis

Continuous variables are presented as the mean or median and categorical variables as *n* (%). Meta-analysis was performed using STATA (StataCorp, TX, Version 15). Hazard ratios (HRs) and risk ratios (RRs) were calculated using the *metan* function. A *P*-value <0.05 was considered significant. The heterogeneity was assessed using an *I*^2^ value to measure variability in observed effect estimates between the studies and heterogeneity was explored with meta-regression in comprehensive meta-analysis software.^8^ Utilizing the unrestricted maximum likelihood assumption, the univariate meta-regression shows the unit change in effect size (i.e. the HR for MCI) standardized to a 10 unit change in predictor variable (e.g. per 10-year increase in age), with associated 95% confidence interval (CI) and *P* value. Separate meta-analyses were performed for (i) cognitive impairment in all AF patients with (ii) a subgroup analysis of those without previous stroke, and (iii) post-stroke cohort; (iv) progression of cognitive impairment; (v) silent cerebral infarction (SCI); and (vi) microbleeds in AF patients. Incidence rate ratios were calculated using follow-up and event data; using the restricted maximum-likelihood estimator random effects model, Poisson-Normal model with log incidence rate as the outcome measure was fitted. Pooled incidence rate was calculated by performing back-transformation of log incidence rates. Where data could not be presented as a meta-analysis, it were reported in a systematic fashion.

## Results

### Search and synthesis of the literature

**Figure 1:**
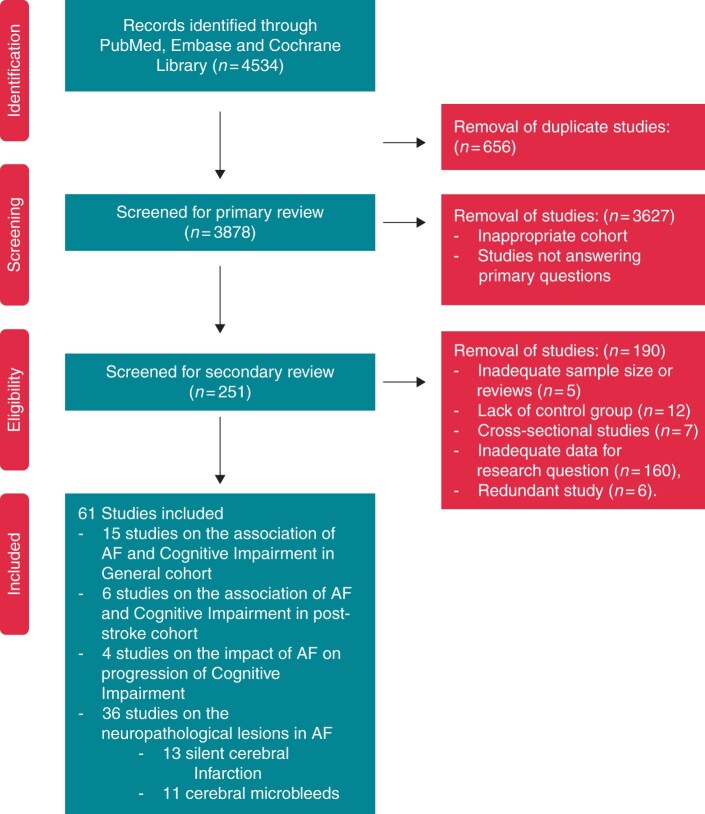
Search strategy.

The online search of PubMed, EMBASE, and Cochrane Library from their inception to 27 March 2021 yielded 4534 citations. Manual searching of references for reviews did not yield additional citations. Subsequently, duplicate citations (656) and citations not conforming to inclusion and exclusion criteria (3627) were excluded from primary review. Two hundred and fifty-one citations were identified for secondary review. After removal of studies with sample size less than previously defined (*n* = 5), the lack of a control group (*n* = 12), cross-sectional studies (*n* = 7), inadequate data for stipulated research questions (*n* = 160), and redundant studies (*n* = 6), 61 studies were included in the final analysis. Of these, 15 studies reported on the association of AF and cognitive impairment in the general population, 6 reported on cognitive impairment in post-stroke population, and 4 on association of AF with progression of cognitive impairment. Using the same pool of retrieved articles, 36 studies reported the neuropathological lesions in the AF population. Of these, 13 reported on the association of AF and SCI, and 11 on cerebral microbleeds (CMB) in AF patients. *Figure 1* provides the consort diagram for the data search.

### Atrial fibrillation and cognitive impairment

#### General population studies

**Figure 2:**
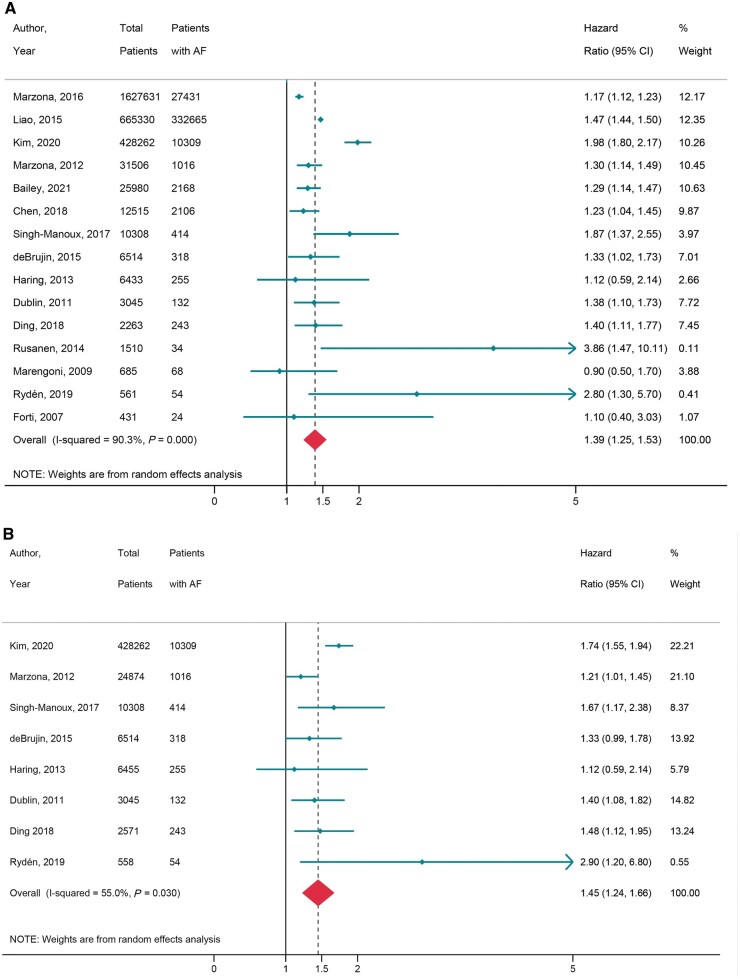
AF and cognitive impairment in (*A*) general population longitudinal studies; and (*B*) general population studies excluding previous history of stroke. AF, atrial fibrillation.

Fifteen prospective population studies,^9–23^ consisting of 2 822 974 patients were analysed. The baseline characteristics of these studies were listed in Supplementary material online, *Table S1*. Thirteen studies^9,^^10,^^12,^^13,^^15–23^ used severe cognitive impairment as their endpoint and two further studies^11,^^14^ used a combination of mild and severe cognitive impairment as their endpoint. The definition of mild and severe cognitive impairment was based on clinical diagnosis in most studies. International Classification of Diseases codes utilized in some studies (Supplementary material online, *Table S1*). The incidence of cognitive impairment amongst participants with and without AF was 17.5% (95% CI 10.9–24.2) and 9.7% (95% CI: 5.1–14.3) events per 1000 person-years of follow-up over a follow-up period of 3.8–25 years, respectively. In the combined analysis (*Figure 2A*), AF was associated with 39% increased risk of cognitive impairment (HR = 1.39, 95% CI 1.25–1.53, *I*^2^ = 90.3%, *P* < 0.001). All studies adjusted for cardiovascular risk factors.

In a pre-specified analysis of studies excluding patients with previous stroke^9–11,^^13,^^14,^^16–18,^^20^ (*n* = 8, *Figure 2B*), AF was associated with 45% increased risk of cognitive impairment (HR = 1.45, 95% CI 1.24–1.66, *I*^2^ = 55.0%, *P* < 0.001).

Only use of anti-coagulants was identified as a source of heterogeneity between effect sizes (coefficient = −0.06, 95% CI −0.11 to 0.02, *P* = 0.005). Other covariate predictors were not identified as a source of heterogeneity; age (coefficient = −0.12, 95% CI −0.26 to 0.01, *P* = 0.08), heart failure (coefficient = −0.01, 95% CI −0.10 to 0.08, *P* = 0.76), hypertension (coefficient = −0.02, 95% CI −0.07 to 0.03, *P* = 0.41), diabetes (coefficient = −0.06, 95% CI −0.22 to 0.09, *P* = 0.43), coronary artery disease (coefficient = −0.07, 95% CI −0.16 to 0.03, *P* = 0.15), previous stroke (coefficient = 0.01, 95% CI −0.10 to 0.11, *P* = 0.99), and length of follow-up (coefficient = 0.05, 95% CI −0.16 to 0.25, *P* = 0.64).

#### Acute stroke cohort

**Figure 3:**
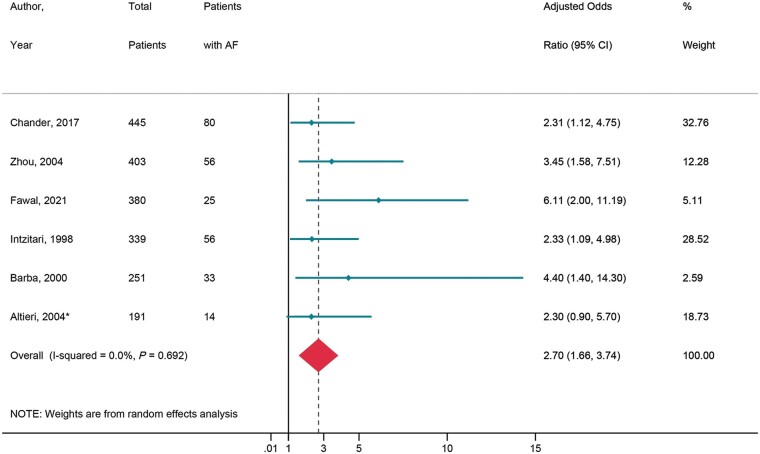
Impact of AF on risk of cognitive impairment after acute stroke. AF, atrial fibrillation.

Six prospective studies^24–29^ consisting of 2009 patients were analysed to evaluate the impact of AF on the risk of cognitive function after acute stroke. The proportion of post-stroke patients with and without AF, developing cognitive impairment was 55% (95% CI: 0.39–0.70) and 21% (95% CI 0.12–0.30), respectively. Overall, AF was associated with a 2.70-fold increased risk of cognitive impairment [adjusted odds ratio (aOR) = 2.70, 95% CI 1.66–3.74, *I*^2^ = 0.0%, *P* < 0.001] (*Figure 3*) over a follow-up period of 0.25–3.78 years.

#### Progression of cognitive impairment

**Figure 4:**
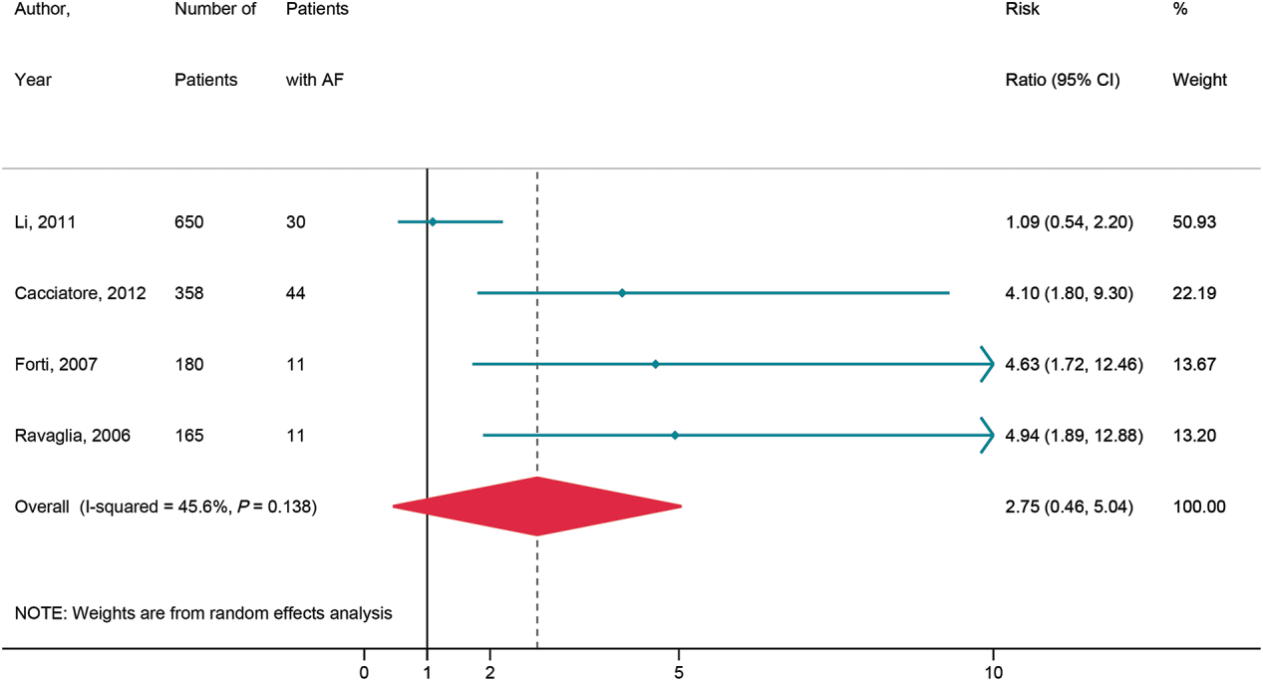
AF and progression of cognitive impairment. AF, atrial fibrillation.

Four studies consisting of 3186 patients^23,^^30–32^ were analysed to assess the impact of AF on the progression from mild to severe cognitive impairment. Overall, there was no significant increase in the risk of progression from mild to severe cognitive impairment [relative risk (RR) = 2.75, 95% CI 0.46–5.04, *I*^2^ = 45.6%) in patients with AF as compared to the control population (*Figure 4*) over 2.8–10 years.

### Atrial fibrillation and neuropathological lesions

Thirty-six studies reported the neuropathological lesions in the AF population. Of these, 32 described findings from brain imaging,^33–64^ while the remaining 4 studies described post-mortem results.^65–68^ The neuropathological changes of the imaging and autopsy studies are listed in *Table 1*. Autopsy studies reported gross and subcortical infarcts in patients with AF.^65–68^ Neuritic plaques^66^ and neurofibrillary tangles,^68^ usually seen with Alzheimer’s disease, were noted to be absent. Imaging studies demonstrated association of decrease in cerebral perfusion,^53,^^54^ and total cerebral volume with AF, after adjusting for infarction,^35,^^49,^^50^ and ApoEε4.^35,^^49^ There was greater decrease in total cerebral volume in permanent as compared to paroxysmal AF.^50^ In addition, decreased frontal lobe and hippocampal volume were associated with AF.^49^ Atrial fibrillation was not associated with Alzheimer’s pattern of ^18^F-FDG PET hypometabolism or PiB uptake (β-amyloid accumulation).^35^

**Table 1:** Neuropathological abnormalities in patients with AF

*Infarction* Autopsy Gross cortical and subcortical infarcts^64–67^ Absence of neuritic plaques^65^ and neurofibrillary tangles^67^ Brain imaging (CT/ MRI) Silent cortical infarction^32–44,^^50^ More in persistent AF as compared to paroxysmal AF^35^ Subcortical infarction^32–34,^^45,^^46^ Severe periventricular white matter lesions/ hyperintensities^45–47^ Cerebral microbleeds^54–63^*Decreased brain volume* Decrease in total cerebral volume Adjusted for infarction,^34,^^48,^^49^ ApoEε4^34,^^48^ Greater decrease in permanent as compared to paroxysmal AF^49^ Decrease frontal lobe volume,^48^ adjusted for ApoEε4 Decrease hippocampal volume Decreased cerebral perfusion^52,^^53^*FDG hypometabolism* Absence of Alzheimer’s disease pattern^34^

AF, atrial fibrillation; CT, computed tomography; FDG, 18F-Deoxyglucose; MRI, magnetic resonance imaging.

#### Silent cerebral infarction in patients with atrial fibrillation

**Figure 5:**
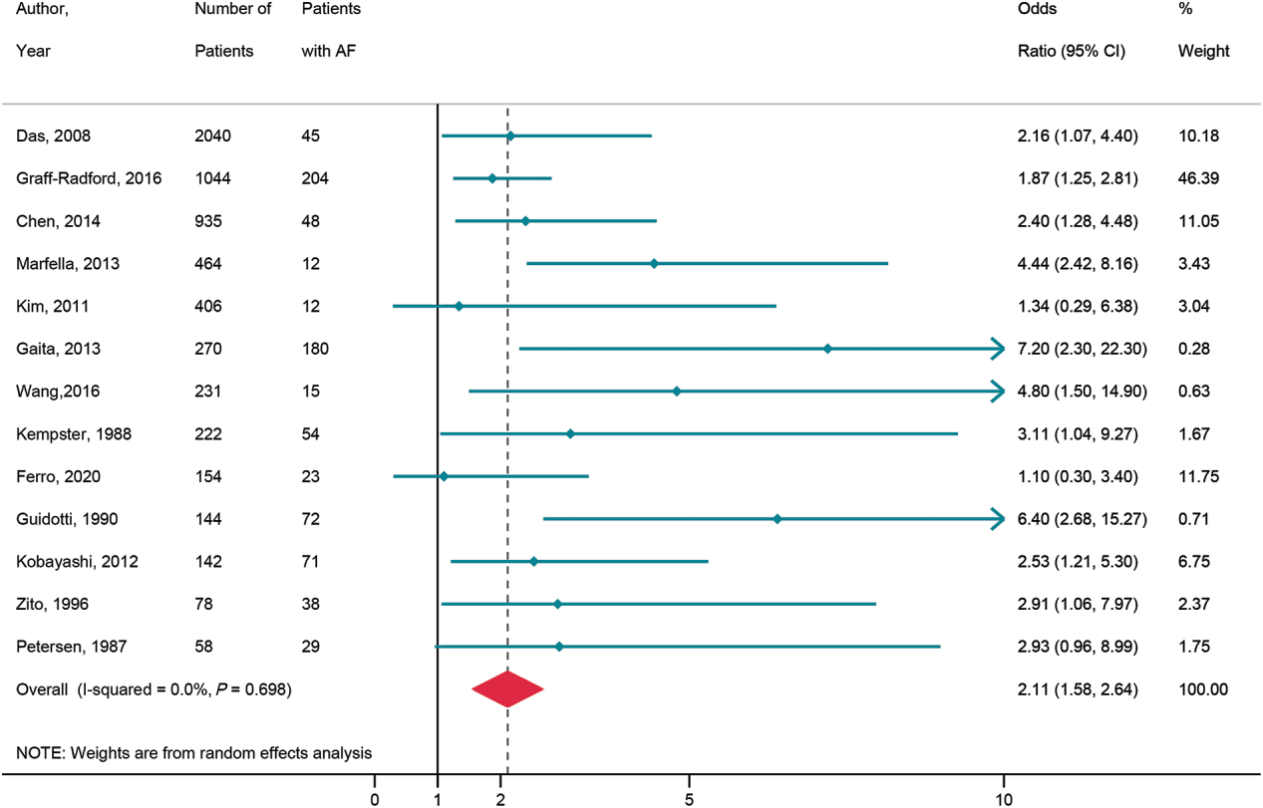
AF and silent cerebral infarction. AF, atrial fibrillation.

Thirteen studies, consisting of 6188 patients [9 magnetic resonance imaging (MRI) studies^33,^^35,^^36,^^39,^^40,^^42–44,^^69^ and 4 computed tomography (CT) studies^37,^^38,^^41,^^45^] were analysed to assess the association of SCI with AF. Supplementary material online, *Table S2* provides the study characteristics and baseline parameters of subjects in the studies. Silent cerebral infarction was defined as hypodense foci on CT studies. The MRI studies used definitions mostly consistent with STRIVE criteria.^70^ Lacunar infarct was defined as focal lesions ≥3 and ≤15 mm and hyperintense on T2-weighted and iso/hypointense on T1-weighted images. Most studies excluded SCI <3 mm due to difficulty in differentiation them from perivascular spaces under 3 mm.^37^ The studies reporting cortical infarct defined them as ≥10 mm in cortical regions. Silent cerebral infarction were noted to be lacunar,^33,^^42,^^44,^^45^ cortical,^35,^^37,^^38^ or both.^36,^^40,^^43^ One study defined cerebral micro-infarcts as <5 mm.^39^ Ten of 13 included studies excluded patients with previous stroke. In the remaining studies, imaging was performed for suspected stroke. Silent cerebral infarction was present in 36.7% of patients (95% CI 14.8–58.7, *I*^2^ = 97.7%) with AF, and 22.9% (95% CI 16.6–29.3, *I*^2^ = 96.6%) without AF. Overall, AF was associated with 2.11-fold increased risk of SCI (OR = 2.11, 95% CI 1.58–2.64, *I*^2^ = 0.0%) (*Figure 5*).

#### Cerebral microbleeds in patients with atrial fibrillation

**Figure 6:**
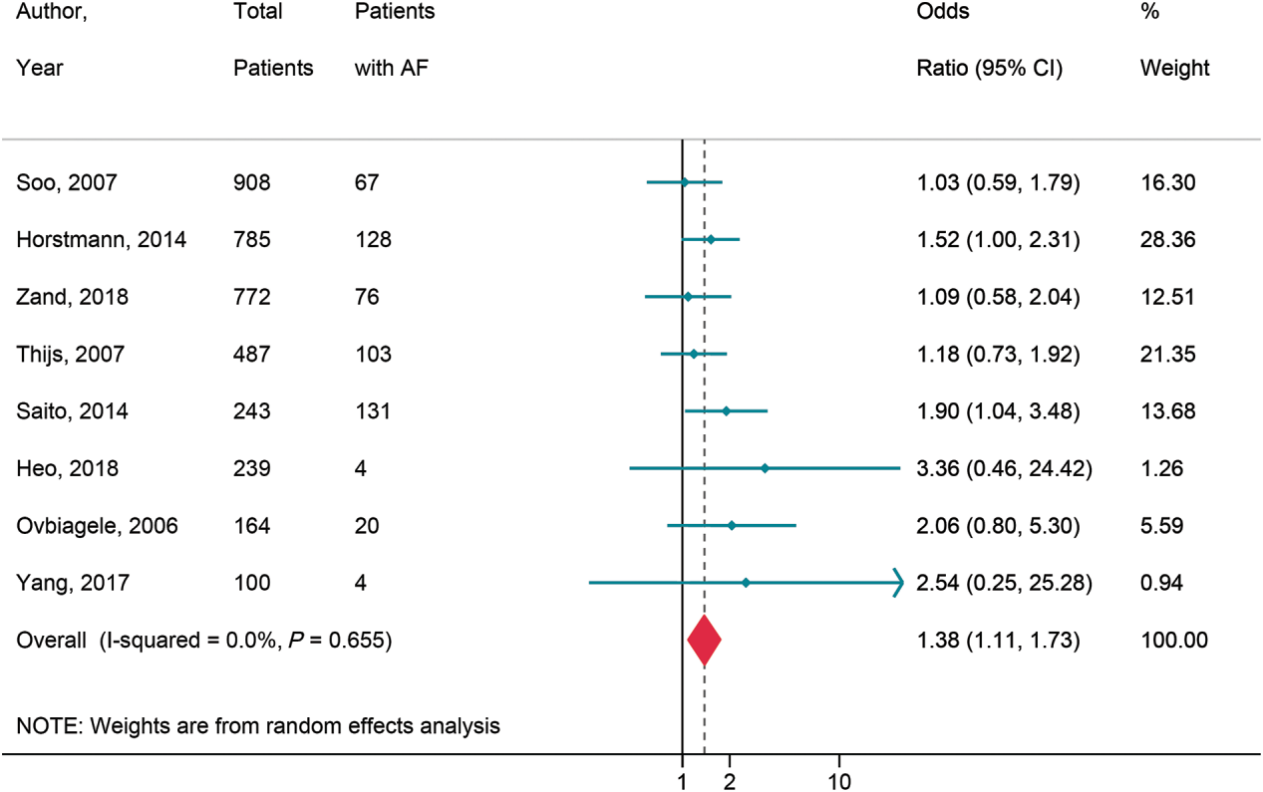
AF and cerebral microbleeds. AF, atrial fibrillation.

A total of 11 studies reported on association of CMB and AF. Supplementary material online, *Table S3* provides the study characteristics and baseline parameters and anticoagulation status of subjects in the studies. Cerebral microbleeds were present in 30.1% of patients (95% CI 23.5–36.7, *I*^2^ = 57.6%) with AF, and 26.5% (95% CI 21.2–31.9, *I*^2^ = 91.4%) without AF. Eight, consisting of 3698 patients, demonstrated a 38% increased likelihood of CMB (OR = 1.38, 95% CI 1.11–1.73, *I*^2^ = 0.0) (*Figure 6*) in patients with AF.^52,^^55–61^ Of these, six examined ischaemic stroke cohorts, one haemorrhagic stroke, and only one evaluated patients with AF undergoing MRI for a neurological indication.

Three studies examined the prognostic role of CMB in AF patients.^62–64^ Overall, presence of CMB at baseline was associated with a 1.7-fold increased risk of composite all-cause death and all-cause stroke at follow-up (*n* = 3, HR = 1.70, 95% CI 1.0–2.403, *I*^2^ = 38.0%).^62–64^

### Study quality and publication bias

All included studies fulfilled the Newcastle Ottawa Score for being representative of the relevant cohort and having adequate sample size. There was low risk of detection and information biases in all studies. Supplementary material online, *Table S4* provides the modified Newcastle Ottawa Score for the included studies. Funnel plots and egger test revealed no significant publication bias in any of the statistical analyses (Supplementary material online, *Figure S1*).

## Discussion

Atrial fibrillation is associated with serious complications ranging from heart failure to debilitating stroke and death. Our systematic review and meta-analyses provide compelling evidence for association of AF with cognitive impairment (*Graphical abstract*). The meta-analyses included only prospective observational studies, which enabled the measurement of events in temporal sequence, providing reliable results. The major findings were

1. The presence of AF is associated with a 39% increased risk of cognitive impairment with a lead time of years to decade(s). The association persisted even after exclusion of patients with previous history of stroke. Furthermore, the presence of AF results in early and 2.7-fold increased risk of cognitive impairment after acute stroke.
2. Atrial fibrillation is associated with increased risk of cerebral small vessel disease, such as white matter lesions, SCI, CMB, and reduced cerebral volume which may represent the plausible link between AF and cognitive impairment.
3. Atrial fibrillation is associated with 38% increased risk of CMB. When present, CMB are associated with increased risk of death and recurrent stroke in patients with AF.

### Atrial fibrillation and cognitive impairment

The Rotterdam Study was the first to describe the association between AF and cognitive impairment.^71^ Although some small studies or studies in elderly cohorts have not shown this association, larger cross-sectional and prospective longitudinal studies have confirmed high risk of cognitive impairment in patients with AF.^6,^^33^ The risk of cognitive decline has also been noted to be dependent on the duration of exposure or when AF is diagnosed earlier than the eighth decade.^6,^^9,^^16^ However, this association is confounded by the presence of shared cardiovascular risk factors, such as hypertension, diabetes mellitus, heart failure, and excess alcohol intake. Some studies have shown a lower risk of dementia in patients with AF on oral anticoagulation providing evidence in favour of the causal association of AF and dementia.^72,^^73^ Similarly, patients managed with rhythm control by catheter ablation may have a lower risk of dementia.^74^ The current meta-analysis restricted itself to prospective studies that had adjusted risk for cardiovascular risk factors, providing strength to the association between AF and cognitive impairment. The lengthy follow-up of prospective studies also suggests a long lead time in absence of clinical stroke. However, the cognitive impairment was more common and accelerated to occur within months to years after acute stroke. The association between AF and progression of cognitive impairment did not achieve significance and may represent lack of adequate power of the analysis.

### Link between atrial fibrillation and cognitive impairment

Atrial fibrillation is an established risk factor for stroke, accounting for up to one-third of the stroke cases in elderly patients. The presence of AF has been associated with large ischaemic lesions secondary to macro-embolism from the left atrium.^66,^^68^ These lesions increase the risk of developing large vessel dementia.^65^ Even in absence of clinical stroke, several mechanisms have been proposed to explain the increased risk of cognitive impairment in patients with AF.

This meta-analysis comprehensively presents the various neuropathological changes associated with AF that could represent a plausible link between AF and cognitive impairment. Silent micro-emboli secondary to thrombogenic and inflammatory state in AF have been proposed to result in not only silent cortical^33–45,^^75^ and but also silent sub-cortical infarction.^33–35,^^46,^^47^ However, associated small vessel disease may contribute to silent sub-cortical infarction. Hypoperfusion secondary to variability in cerebral blood flow and cerebral vascular rhythm may result in ischaemia or impairment of flowing blood’s ability to remove micro emboli from the vessels which results in embolic infarction.^76^ In addition, the burden of cerebral small vessel disease, such as white matter lesions, increases with a chronic reduction in cerebral blood flow secondary to persistent AF.^36^ Although certain studies suggest an association with Alzheimer’s disease, autopsy, and positron emission tomography (PET) imaging have demonstrated an absence of neuritic plaques,^66^ neurofibrillary tangles,^68^ and Alzheimer’s disease pattern.^35^ Furthermore, decreased cerebral perfusion^54^ may explain reduction in cerebral volume in patients with AF, even after adjustment for infarction^35,^^49,^^50^ and ApoEε4.^35,^^49^ The meta-analysis also confirmed that CMB, a marker of cerebral small vessel disease, are seen more often in patients with AF. Recent data suggest that novel oral anticoagulants may not increase risk of CMB as they do not cross the blood brain barrier.^77^ CMB have been shown to be associated with greater risk of cerebral haemorrhage and stroke. The risk for recurrent ischaemic stroke is greater than the risk for cerebral haemorrhage even in patients on oral anticoagulants.^62^ Our meta-analysis confirmed that CMB are associated with increased risk of death and all-cause stroke in patients with AF.

To summarize, cerebral small vessel disease, clinical, and subclinical infarction are associated with AF. We hypothesize that these changes when superimposed on the concomitant cerebral small vessel disease associated with comorbid conditions such as hypertension and diabetes predispose patients to cognitive impairment (*Graphical abstract*).

### Strengths and limitations

The results of the current study were compiled using meta-analysis of primarily observational data. However, the technique of meta-analysis is well accepted in the literature to aggregate results from observational data, to facilitate synthesis of available evidence and the infarction were also heterogenous in the included studies. Although previous meta-analyses have shown similar association with cognitive function,^78,^^79^ this updated meta-analyses included only prospective longitudinal studies only and demonstrated a robust relationship despite adjusting for cardiovascular risk factors. In addition, this meta-analysis highlights the potential timeline of cognitive impairment based on the length of follow-up. The study also systematically reviews the association of gamut of neuropathological changes in patients with AF. The impact of oral anticoagulation on cognitive impairment in patients with AF was not evaluated as it way beyond the scope of the meta-analyses. This meta-analysis provides important information that will be useful to estimate sample size and design further prospective studies to inform on measures that may reduce risk of cognitive impairment due to AF.

## Conclusion

Atrial fibrillation is associated with increased risk of cognitive impairment. Clinical and silent brain infarction, cerebral small vessel disease, and cerebral atrophy secondary to cardioembolism and cerebral hypoperfusion may represent the plausible link in absence of clinical stroke. Further prospective randomized control trials are essential to further the understanding of the mechanisms of cognitive impairment and to develop strategies to prevent cognitive impairment in patients with AF.

## Supplementary material

Supplementary material is available at *Europace* online.

##  

### Funding

D.A.M. was supported by the Post-doctoral Research Fellowship from the University of Adelaide. A.D.E. was supported by an Early Career fellowship from the National Heart Foundation (NHF). D.L. was supported by a Beacon Research Fellowship from the University of Adelaide. D.H.L. was supported by the Robert J. Craig Lectureship from the University of Adelaide. R.M. was supported by the Mid-Career Fellowship from the Hospital Research Fund. This study was supported by funds from the University of Adelaide. Several of the authors are employees of the University of Adelaide. The sponsor has had no direct involvement in the management or outcomes of the study.

## Supplementary Material

euac003_Supplementary_Data

## Data Availability

Data are available on request to the corresponding author.
