# Supplementary material for: Predictive role of atrial fibrillation in cognitive decline: a systematic review and meta-analysis of 2.8 million individuals
Source: Europace. 2022 Jan 21;24(8):1229–39. doi: 10.1093/europace/euac003 (PMC9435641; doi:10.1093/europace/euac003)

**Predictive Role of Atrial Fibrillation in Cognitive Decline: A Systematic Review and Meta-Analysis**

***Short title:*** *AF and cognitive impairment*

Yu Han Koh;^1*^ Leslie Z.W. Lew;^1*^ Kyle B. Franke;^1^ Adrian D. Elliott, PhD;^1^ Dennis H. Lau, MBBS, PhD;^1-2^ Anand Thiyagarajah, MBBS;^2^ Dominik Linz, MD, PhD;^1^ Margaret Arstall, MBBS, PhD;^1,3^ Phillip J. Tully, M.Psych(Clin), PhD;^1^ Bernhard T. Baune, MD, PhD;^4-6^ Dian A. Munawar, MD, PhD;^1,2,7^ Rajiv Mahajan, MD, PhD.^1,3^

**From:** ^1^The University of Adelaide, Australia; ^2^Royal Adelaide Hospital, Adelaide, Australia; ^3^Lyell McEwin Hospital, Adelaide, Australia; ^4^Department of Psychiatry, University of Melbourne, Melbourne Australia; ^5^The Florey Institute of Neuroscience and Mental Health, The University of Melbourne, Parkville, VIC, Australia; ^6^Department of Psychiatry, University of Münster, Germany; ^7^Department of Cardiology and Vascular Medicine, University of Indonesia, Jakarta, Indonesia.

* First equal

**Supplementary Methods S1: Search Strategy**

("Major Neurocognitive Disorder"[All Fields] OR "Dementia"[All Fields] OR "Cognitive Impairment"[All Fields] OR "Alzheimer's Disease"[All Fields] OR “Vascular Dementia” [All Fields] "Silent Cerebral Infarct"[All Fields] OR "Silent Cerebral Infarction"[All Fields] OR "Silent Brain Infarction"[All Fields] OR "Silent Brain Infarct"[All Fields] OR "Cerebral Microbleed"[All Fields] OR "Cerebral Microhemorrhage"[All Fields] OR "Brain Microbleed"[All Fields] OR “Brain Microhemorrhage”) AND ("Atrial Fibrillation"[All Fields])

**Supplementary Table 1: Baseline parameters of the prospective studies evaluating association of AF and cognitive decline**

| **Author, Year** | **Follow up (Years)** | **Previous stroke**  **excluded** | **Definition of cognitive impairment (primary outcome)** | **Number of patients** | **AF status** | **Age ± SD/(IQR) (Years)** | **CHF (%)** | **HT (%)** | **DM (%)** | **Previous stroke (%)** | **CAD (%)** | **Anticoagulation (%)** |
| --- | --- | --- | --- | --- | --- | --- | --- | --- | --- | --- | --- | --- |
| **General Population Cohort Studies** | | | | | | | | | | | | |
| deBruijn, 2015 | 20 | Yes | MDT diagnosis | 6194 | No AF | 68.3 ± 8.5 | 2.5 | NR | 9.0 | 0 | 7.9 | 22.4 |
|  |  |  |  | 318 | AF | 75.7 ± 8.1 | 18.8 | NR | 20.1 | 0 | 18 | 27.4 |
| Dublin, 2011 | 14 | Yes | MDT diagnosis | 2913 | No AF | 74.2 (70–79) | 2.7 | 31.9 | 9.3 | 0 | 18 | 0.9 |
|  |  |  |  | 132 | AF | 76.5 (71–82) | 18.2 | 40.9 | 11.5 | 0 | 36.4 | 40.9 |
| Haring, 2013 | 8.6 | Yes | Physician diagnosis based on DSM IV | 6178 | No AF | NR | NR | NR | NR | 0 | NR | NR |
|  |  |  |  | 255 | AF | NR | NR | NR | NR | 0 | NR | NR |
| Kim, 2020 | 9 | No | ICD-10 codes | 419279 | No AF | 55.5 ± 9.1 | 2.4 | 22.6 | 7.5 | 0 | 0.8 | 0.04 |
|  |  |  |  | 10983 | AF | 61.7 ± 9.9 | 8.9 | 40.5 | 12.4 | 0 | 2.5 | 0.44 |
| Liao, 2015 | 15 | No | ICD-9  codes | 332665 | No AF | 70.3 ± 13.0 | 9.2 | 46.1 | 19.8 | 17.2 | NR | 0.6 |
|  |  |  |  | 332665 | AF | 70.3 ± 13.0 | 40.2 | 67.4 | 27.8 | 32.3 | NR | 10 |
| Marengoni, 2009 | 6 | Yes | Physician diagnosis based on DSM IV | 617 | No AF | NR | NR | NR | NR | 0 | NR | NR |
|  |  |  |  | 68 | AF | NR | NR | NR | NR | 0 | NR | NR |
|  |  |  |  | 10161 | AF | 68.1 ± 12.2 | 1.8 | 44.3 | 15.6 | 3.2 | 7.6 | NR |
| Marzona, 2016 | 10 | No | ICD-9 code or dementia drug prescription at discharge | 1600200 | No AF | 75.2±7.1 | 1.4 | 61.8 | 12 | NR | 4.3 | 3.3 |
|  |  |  |  | 27431 | AF | 78.4 ± 7.2 | 27.3 | 86.8 | 18.1 | NR | 28.7 | 36.4 |
| Singh-Manoux, 2017 | 5 | No | Electronic health record diagnosis | 7014 | No AF | 55.5 (6.0) | 0.04 | 28 | 4.3 | NR | NR | NR |
|  |  |  |  | 414 | AF | 58.8 (5.9) | 0 | 43.7 | 5.6 | NR | NR | NR |
| Marzona, 2012 | 5 | No | Clinical diagnosis or MMSE | 29916 | No AF | 66.3 ± 7.2 | NR | 70.1 | 37.3 | NR | NR | 5.6 |
|  |  |  |  | 1016 | AF | 70.3 ± 6.9 | NR | 74.8 | 6.1 | NR | NR | 62.4 |
| Ryden, 2019 | 10.4 | Yes | NPR or DSM III | 54 | No AF | 70 | 2.2 | 83.0 | 6.4 | 0 | 8.5 | NR |
|  |  |  |  | 507 | AF | 70 | 13.0 | 77.8 | 11.1 | 0 | 27.8 | NR |
| Ding, 2018 | 5.8 | No | MDT diagnosis | 2442 | No AF | 72.3±10.3 | 6.2 | 74.0 | 8.9 | 3.4 | 12.1 | NR |
|  |  |  |  | 243 | AF | 80.9±9.4 | 42.0 | 86.4 | 14.0 | 13.2 | 31.3 | NR |
| Bailey, 2021 | 8.06 | Yes | Six item screener | 23812 | No AF | 64.1±9.2 | NR | 50.4 | 20.0 | NR | 34.8 | NR |
|  |  |  |  | 2168 | AF | 67.2±9.7 | NR | 63.8 | 25.2 | NR | 14.6 | NR |
| Chen, 2018 | 20.2 | No | MDT diagnosis | 2106 | No AF | 56.4±5.6 | 4.0 | 45.0 | 13.0 | 1.0 | 5.0 | 3.0 |
|  |  |  |  | 10409 | AF | 59.3±5.4 | 8.0 | 29.0 | 20.0 | 2.0 | 10.0 | 30.0 |
| Forti, 2007 | 3.8 | No | Geriatric physicians (x3) | 159 | No AF | NR | NR | NR | NR | NR | NR | NR |
|  |  |  |  | 11 | AF | NR | NR | NR | NR | NR | NR | NR |
| Rusanen, 2014 | 25.5 | Yes | MDT + DSM IV | 1510 | Both | 50.3±6.0 | NR | NR | 10.5 | 7.2 | NR | NR |
| **Acute Stroke Cohort Studies** | | | | | | | | | | | | |
| Barba, 2000 | 0.25 | - | DSM IV | 218 | Both | NR | 5.6 | 59.8 | NR | 12.4 | NR | NR |
|  |  |  |  | 33 |  |  |  |  |  |  |  |  |
| Intzitari, 1998 | 1 | - | ICD-10 criteria | 282 | Both | 71.8±11.3 | NR | 52.6 | 24.9 | 13.8 | NR | NR |
|  |  |  |  | 56 |  |  |  |  |  |  |  |  |
| Zhou, 2004 | 0.25 | - | DSM IV | 377 | Both | NR | 18.7 | 66.6 | 17.7 | 26.0 | NR | NR |
|  |  |  |  | 56 |  |  |  |  |  |  |  |  |
| Chander, 2017 | 0.28 | - | MMSE<26 | 365 | No AF | 59.1±11.9 | NR | 73.7 | 45.0 | 15.6 | 15.3 | NR |
|  |  |  |  | 80 | AF | 71.4±10.2 | NR | 87.5 | 38.4 | 23.8 | 39.7 | NR |
| Fawal, 2021 | 0.50 | - | MMSE<25 | 355 | Both | NR | NR | 57.9 | 39.5 | NR | NR | NR |
|  |  |  |  | 25 |  |  |  |  |  |  |  |  |
| Altieri, 2004 | 3.78 | - | ICD-10 criteria | 177 | Both | NR | NR | 71.7 | 20.9 | 34.6 | NR | NR |
|  |  |  |  | 14 |  |  |  |  |  |  |  |  |
| **Progression of Cognitive Impairment Studies** | | | | | | | | | | | | |
| Cacciatore | 10 | Yes | DSM-IV+MDT | 314 | No AF | 73.7±7.1 | 10.9 | NR | 15.3 | 0.0 | 13.7 | NR |
|  |  |  |  | 44 | AF | 74.4±6.9 | 34.2 | NR | 13.6 | 0.0 | 27.3 | NR |
| Forti, 2007 | 3.8 | No | Geriatric physicians (x3) | 169 | Both | NR | NR | NR | NR | NR | NR | NR |
|  |  |  |  | 11 |  |  |  |  |  |  |  |  |
| Li, 2011 | 5 | No | DSM-IV | 620 | Both | NR | NR | 33.2 | 20.9 | NR | NR | NR |
|  |  |  |  | 30 |  |  |  |  |  |  |  |  |
| Ravaglia, 2006 | 2.8 | No | Clinical diagnosis (AAN guidelines) | 154 | Both | NR | NR | 38.2 | 9.7 | 6.1 | NR | NR |
|  |  |  |  | 11 |  |  |  |  |  |  |  |  |

AAN: American Academy of Neurology; AF: Atrial fibrillation; CAD: Coronary artery disease; CHF: Congestive heart failure; DM: Diabetes mellitus; HT: Hypertension; MDT: Multi-disciplinary team; NPR: National patient register; NR: Not reported; SD: Standard Deviation

**Supplementary Table 2: Study characteristics and baseline parameters of subjects in studies evaluating silent cerebral infarction and AF**

| **Study** | **Study characteristics** | **Previous Stroke excluded** | **Imaging Modality** | **Cognitive impairment assessment** | **Statistical Analysis- adjustment for risk factors** | **Groups** | **Age ± SD/(CI) (Years)** | **CHF (%)** | **HT (%)** | **DM (%)** | **CAD (%)** | **Anticoagulation (%)** |
| --- | --- | --- | --- | --- | --- | --- | --- | --- | --- | --- | --- | --- |
| Chen 2014 (Atherosclerosis Risk in  Communities Study) | Prospective study, 935 community subjects | Yes | MRI | DSS and WF tests* | Yes. 2 models | AF | 63.2±4.3 | 0.1 | 54 | 15 | 2.3 | NR |
|  |  |  |  |  |  | No AF | 61.4±4.3 | 0.1 | 42 | 13 | 10 | NR |
| Graff-Radford 2016 (Mayo Clinic Study of Aging) | Prospective longitudinal study, 1044 community subjects | Yes | MRI | MCI, Mayo criteria | Yes. Age, sex, APOE ε4 carrier status and education | AF | 80 [76-83] | NR | 88 | 27 | 66 | 27 |
|  |  |  |  |  |  | No AF | 77 [73-82] | NR | 71 | 17 | 37 | 2 |
| Gaita 2013 | Prospective cohort,  270 consecutive patients referred to cardiology | Yes | MRI | RBANS** | Yes. Age, CHA2DS2-Vasc score and OAC | AF | 61.2 ±10.9 | NR | 52 | 7.8 | 3.3 | 87 |
|  |  |  |  |  |  | No AF | 59.7±13.1 | NR | 50 | 5.6 | 4.4 | 0 |
| Guidotti 1990 | 72 patients with chronic AF were compared to control group, affected  by muscle-tensive headache. | Yes | CT | No | Age and sex matched | AF | 68 | NR | 33 | 11 | 0 | NR |
|  |  |  |  |  |  | No AF | 68 | NR | 22 | 6 | 0 | NR |
| Kobayashi 2012 | 71 consecutive patients with NVAF and 71 sex-and age-matched controls  with sinus rhythm undergoing MRI | Yes | MRI | No | No | AF | 74.4±9.9 | NR | 63 | 28 | NR | 31 |
|  |  |  |  |  |  | No AF | 73.7±8.2 | NR | 58 | 27 | NR | 1.4 |
| Kempster 1998 | Consecutive patient with chronic AF and suspected stroke and CT head | No | CT | No | No | AF | NR | NR | NR | NR | NR | NR |
|  |  |  |  |  |  | No AF | NR | NR | NR | NR | NR | NR |
| Marfella 2013 | Prospective, longitudinal observational study studying association of subclinical AF with stroke in diabetics, 460 diabetics subjects and 240 healthy controls | Yes | MRI | No | Yes | AF (Subclinical) | 50±5 | 0 | 0 | 100 | 0 | 0 |
|  |  |  |  |  |  | No AF | 51±9 | 0 | 0 | 0 | 0 | 0 |
| Petersen 1987 Controls from Copenhagen  City Heart Study | 30 consecutive AF patients; and 30 healthy controls | Yes | CT | No | No | AF | NR | 70 | 73 | NR | NR | NR |
|  |  |  |  |  |  | No AF | NR | 0 | 0 | 0 | 0 | 0 |
| Zito 1996 | 38 patients with AF compared with 40 controls | Yes | CT | MMSE | No | AF | 80.6 | NR | NR | NR | NR | NR |
|  |  |  |  |  |  | No AF | 80.4 | NR | NR | NR | NR | NR |
| Das 2008 (Framingham Offspring Study) | Prospective, longitudinal cohort study, 2040 community subjects | Yes | MRI | No | Yes. Age and sex | SCI | 64±9 | NR | 50 | 12.7 | 11.8 | NR |
|  |  |  |  |  |  | No SCI | 62 ±9 | NR | 35 | 8.2 | 7 | NR |
| Ferro 2020 (Heart-Brain Connection Consortium) | Prospective heart failure cohort, 154 heart failure subjects | No (12% vs 4%) | MRI | MMSE***, Neuropsychological tests | Yes. Age, sex and vascular risk factors | SCI | 71.3 ± 8.4 | 100 | 62 | 23 | 69 | 27 |
|  |  |  |  |  |  | No SCI | 69.1 ± 10.3 | 100 | 50 | 17 | 50 | 32 |
| Kim 2011 | Retrospective cohort, 406 subjects with MRI brain | Yes | MRI | No | No | SCI | 67.1±9.8 | NR | 68.8 | 31.3 | 4.7 | NR |
|  |  |  |  |  |  | No SCI | 63.9 ±10.7 | NR | 42.1 | 20.8 | 3.2 | NR |
| Wang 2016 (STRIDE study) | Prospective, cortical SCI assessed in 231 subjects with suspected stroke | No  (29% vs 16%) | MRI | MoCA****  Serial assessment over 5 years |  | SCI | 68.7±8.7 | NR | 58.8 | 44 | NR | NR |
|  |  |  |  |  |  | No SCI | 66.9±12.5 | NR | 68 | 34 | NR | NR |

AF: Atrial Fibrillation; SCI: Silent Cerebral Infraction; CAD: Coronary artery disease; CHF: Congestive heart failure; DM: Diabetes mellitus; HT: Hypertension; NR: Not reported; * Digit Symbol Substitution [DSS] and the Word Fluency [WF] test); *RBANS: Repeatable Battery for the Assessment of Neuropsychological Status; *** MMSE: Mini Mental Status Examination; ****MoCA: Montreal Cognitive Assessment

**Supplementary Table 3: Study characteristics and baseline parameters of subjects in studies on cerebral microbleeds**

| **Study** | **Study characteristics** | **Previous Stroke excluded** | **Cognitive impairment assessment** | **Statistical Analysis- adjustment for risk factors** | **Groups** | **Age Mean ±SD/Median [IQR] (Years)** | **CHF (%)** | **HT (%)** | **DM (%)** | **CAD (%)** | **Oral Anticoagulation (%)** |
| --- | --- | --- | --- | --- | --- | --- | --- | --- | --- | --- | --- |
| Heo, 2018 | Prospective stroke/TIA cohort of 235 patients | No | No | No | CMB | 66.5±9.7 | NR | 69.2 | 23.1 | NR | NR |
|  |  |  |  |  | No CMB | 61.3±12.6 | NR | 55.0 | 20.6 | NR | NR |
| Horstmann, 2014 | Prospective stroke/TIA cohort of 785 patients | No | No | No | CMB | 72 [64-78] | NR | 86.6 | 30.1 | NR | 9.7 |
|  |  |  |  |  | No CMB | 64 [52-73] | NR | 73.0 | 20.3 | NR | 5.2 |
| Ovbiagele, 2006 | Prospective stroke/TIA cohort of 164 patients | No | N/A | No | CMB | 78* | NR | 73.7 | NR | 26.3 | 3.51 |
|  |  |  |  |  | No CMB | 73* | NR | 63.6 | NR | 18.7 | 7.5 |
| Saito, 2014 | Prospective cohort study: 131 AF vs 112 controls | Yes | N/A | No | AF | 69.4±9.2 | NR | 55.7 | 27.5 | NR | 64.1 |
|  |  |  |  |  | Control | 69.0±9.5 | NR | 38.4 | 14.3 | NR | 0.0 |
| Soo, 2007 | Prospective stroke/TIA cohort of 908 patients | No | No | No | CMB | 71.2±10 | NR | 79.4 | 32.5 | 7.5 | 2.8 |
|  |  |  |  |  | No CMB | 67.3±11.8 | NR | 63.7 | 30.2 | 8.7 | 3.4 |
| Thijs, 2007 | Prospective stroke/TIA cohort of 487 patients | No | No | No | CMB | 73±9 | NR | 69.0 | 16.0 | 25.0 | 36.0 |
|  |  |  |  |  | No CMB | 72±10 | NR | 63.0 | 20.0 | 22.0 | 30.0 |
| Yang 2017 | Prospective intracerebral hemorrhage cohort of 100 patients | No | No | No | CMB | 62.7±13.5 | NR | 58.2 | 20.0 | NR | NR |
|  |  |  |  |  | No CMB | 58.0±11.0 | NR | 62.2 | 13.3 | NR | NR |
| Zand, 2018 | Prospective study of 772 patients with acute ischaemic stroke | No | N/A | No | CMB | 66.0±13.3 | NR | 90.3 | 43.0 | NR | NR |
|  |  |  |  |  | No CMB | 61.3±14.3 | NR | 75.8 | 33.1 | NR | NR |

Age *Median age

**Supplementary Table 4**: Study quality assessed by modified Newcastle Ottawa Scale (NOS)

**Supplementary Table 4A:** Study quality assessed by modified Newcastle-Ottawa Score: AF and cognitive impairment in general population

| **Author, Year** | **Source population representative** | **Performance bias** | | **Detection bias** | | **Information bias** | |
| --- | --- | --- | --- | --- | --- | --- | --- |
|  |  | **Sample size adequate** | **Adjustment for Other factors** | **Statistical methods appropriate** | **Missing data** | **Methodology of outcome- explicit** | **Objective assessment** |
| deBruijn, 2015^8^ | 3 | 3 | 3 | 3 | 3 | 3 | 3 |
| Dublin, 2011^9^ | 2 | 3 | 3 | 3 | 3 | 2 | 3 |
| Haring, 2013^10^ | 3 | 3 | 3 | 3 | 3 | 3 | 3 |
| Kim, 2020 | 3 | 3 | 3 | 3 | 3 | 2 | 2 |
| Liao, 2015^11^ | 3 | 3 | 3 | 3 | 3 | 3 | 3 |
| Marengoni, 2009^12^ | 3 | 3 | 3 | 3 | 3 | 3 | 3 |
| Marzona, 2016^14^ | 3 | 3 | 3 | 3 | 3 | 3 | 2 |
| Singh-Manoux, 2017^15^ | 3 | 3 | 3 | 3 | 2 | 3 | 3 |
| Marzona, 2012^13^ | 2 | 3 | 3 | 3 | 3 | 3 | 3 |
| Ryden, 2019 | 3 | 3 | 3 | 3 | 3 | 3 | 3 |
| Ding, 2018 | 3 | 3 | 3 | 3 | 3 | 3 | 3 |
| Bailey, 2021 | 3 | 3 | 3 | 3 | 3 | 3 | 3 |
| Chen, 2018 | 3 | 3 | 3 | 3 | 3 | 3 | 3 |
| Forti, 2007 | 3 | 3 | 2 | 3 | 2 | 3 | 3 |
| Rusanen, 2014 | 3 | 3 | 3 | 3 | 2 | 2 | 3 |

Score of 0-3 represent increasing risk of bias with 0 representing high risk and 3 representing low risk

**Supplementary Table 4B:** Study quality assessed by modified Newcastle-Ottawa Score: AF and cognitive impairment in post-stroke cohort

| **Study** | **Source population representative** | **Performance bias** | | **Detection bias** | | **Information bias** | |
| --- | --- | --- | --- | --- | --- | --- | --- |
|  |  | **Sample size adequate** | **Adjustment for Other factors** | **Statistical methods appropriate** | **Missing data** | **Methodology of outcome- explicit** | **Objective assessment** |
| Barba, 2000 | 3 | 3 | 3 | 3 | 3 | 3 | 3 |
| Intzitari, 1998 | 3 | 3 | 3 | 3 | 3 | 3 | 3 |
| Zhou, 2004 | 3 | 3 | 3 | 3 | 3 | 3 | 3 |
| Chander, 2017 | 3 | 3 | 3 | 3 | 3 | 3 | 3 |
| Fawal, 2021 | 3 | 3 | 3 | 3 | 3 | 3 | 3 |
| Altieri, 2004 | 2 | 3 | 3 | 3 | 3 | 3 | 3 |

Score of 0-3 represent increasing risk of bias with 0 representing high risk and 3 representing low risk

**Supplementary Table 4C:** Study quality assessed by modified Newcastle-Ottawa Score: AF and progression of cognitive impairment

| **Study** | **Source population representative** | **Performance bias** | | **Detection bias** | | **Information bias** | |
| --- | --- | --- | --- | --- | --- | --- | --- |
|  |  | **Sample size adequate** | **Adjustment for Other factors** | **Statistical methods appropriate** | **Missing data** | **Methodology of outcome- explicit** | **Objective assessment** |
| Cacciatore, 2012 | 3 | 3 | 3 | 3 | 3 | 3 | 3 |
| Forti, 2007 | 3 | 3 | 3 | 3 | 2 | 2 | 3 |
| Li, 2011 | 2 | 3 | 3 | 3 | 3 | 3 | 3 |
| Ravaglia, 2006 | 3 | 3 | 3 | 3 | 3 | 3 | 2 |

Score of 0-3 represent increasing risk of bias with 0 representing high risk and 3 representing low risk

**Supplementary Table 4D:** Study quality assessed by modified Newcastle-Ottawa Score: Silent Cerebral Infarction in AF

| **Study** | **Source population representative** | **Performance bias** | | **Detection bias** | | **Information bias** | |
| --- | --- | --- | --- | --- | --- | --- | --- |
|  |  | **Sample size adequate** | **Adjustment for Other factors** | **Statistical methods appropriate** | **Missing data** | **Methodology of outcome- explicit** | **Objective assessment** |
| Ferro 2020 | 3 | 3 | 3 | 3 | 3 | 3 | 3 |
| Graff-Radford 2016 | 3 | 3 | 3 | 3 | 3 | 3 | 2 |
| Gaita 2013 | 3 | 3 | 3 | 3 | 3 | 3 | 3 |
| Chen 2014 | 3 | 3 | 3 | 3 | 3 | 3 | 3 |
| Wang 2016 | 2 | 3 | 3 | 3 | 3 | 3 | 3 |
| Das 2008 | 3 | 3 | 3 | 3 | 2 | 3 | 3 |
| Kim 2011 | 3 | 3 | 3 | 3 | 3 | 3 | 3 |
| Marfella 2013 | 2 | 3 | 3 | 3 | 3 | 3 | 3 |
| Kobayashi 2012 | 3 | 2 | 3 | 3 | 3 | 3 | 3 |
| Kempster 1998 | 3 | 2 | 2 | 2 | 2 | 3 | 3 |
| Petersen 1987 | 3 | 2 | 3 | 2 | 3 | 2 | 3 |
| Guidotti 1990 | 2 | 2 | 3 | 3 | 2 | 3 | 3 |
| Zito 1996 | 3 | 2 | 3 | 3 | 3 | 3 | 2 |

Score of 0-3 represent increasing risk of bias with 0 representing high risk and 3 representing low risk

**Supplementary Table 4E:** Study quality assessed by modified Newcastle-Ottawa Score: Microbleeds prevalence in AF

| **Study** | **Source population representative** | **Performance bias** | | **Detection bias** | | **Information bias** | |
| --- | --- | --- | --- | --- | --- | --- | --- |
|  |  | **Sample size adequate** | **Adjustment for Other factors** | **Statistical methods appropriate** | **Missing data** | **Methodology of outcome- explicit** | **Objective assessment** |
| **Heo, 2018** | 3 | 3 | 3 | 3 | 3 | 3 | 3 |
| **Horstmann, 2014** | 3 | 3 | 3 | 3 | 3 | 3 | 3 |
| **Ovbiagele, 2006** | 3 | 3 | 3 | 3 | 3 | 3 | 3 |
| **Saito, 2014** | 3 | 3 | 3 | 3 | 3 | 3 | 3 |
| **Soo, 2007** | 3 | 3 | 3 | 3 | 3 | 3 | 3 |
| **Thijs, 2007** | 3 | 3 | 3 | 3 | 3 | 3 | 3 |
| **Yang 2017** | 3 | 3 | 3 | 3 | 3 | 3 | 3 |
| **Zand, 2018** | 3 | 3 | 3 | 3 | 3 | 3 | 3 |

Score of 0-3 represent increasing risk of bias with 0 representing high risk and 3 representing low risk

**Supplementary figure 1. Funnel plot and Egger test**

**Supplementary figure 1a: Funnel plot and Egger test for association of AF with cognitive impairment (Figure 2a)**

Egger test: p=0.858


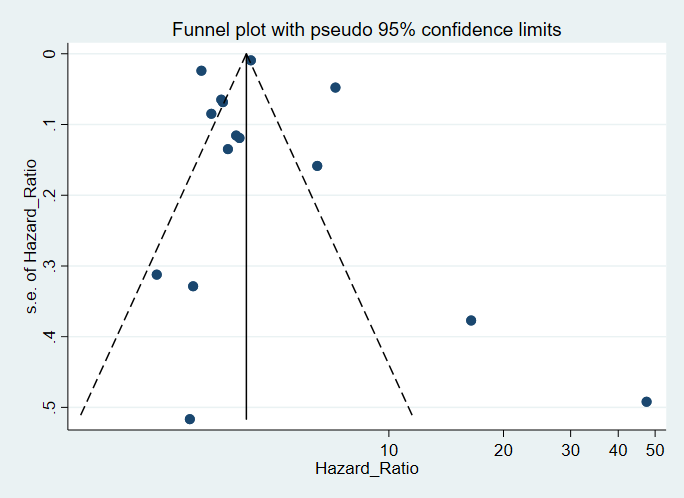


**Supplementary figure 1b: Funnel plot and Egger test for association of AF with cognitive impairment with no past stroke (Figure 2b)**

Egger test: p=0.646


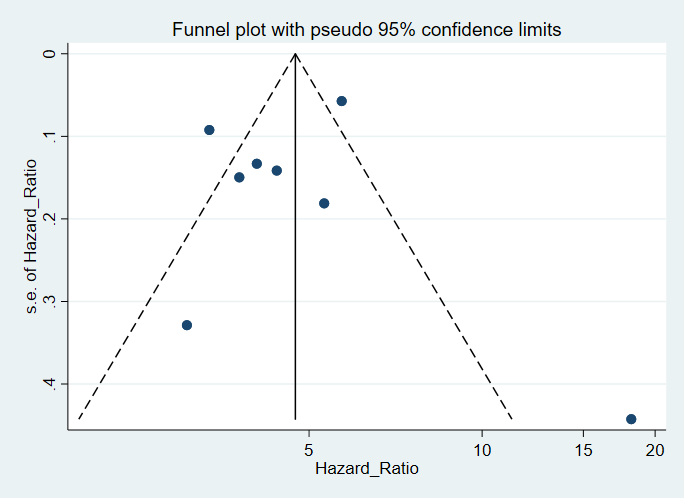


**Supplementary figure 1c: Funnel plot and Egger test for post-stroke cognitive impairment (Figure 3)**

Egger test: p=0.617


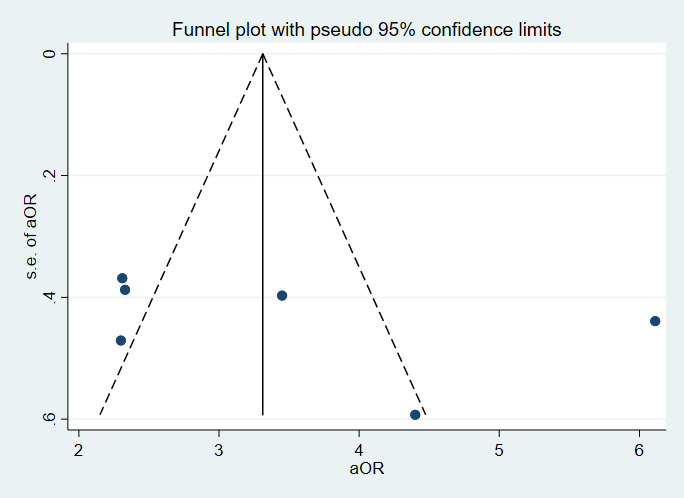


**Supplementary figure 1d: Funnel plot and Egger test for progression of mild cognitive impairment (Figure 4)**

Egger test: p=0.815


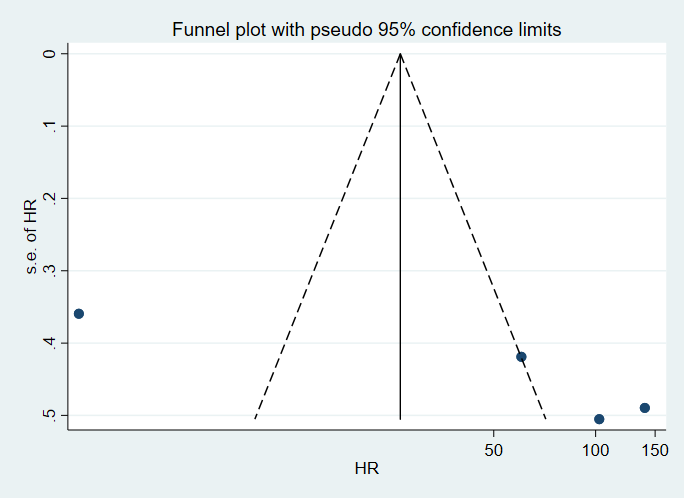


**Supplementary figure 1e: Funnel plot and Egger test for prevalence of silent cerebral infarcts**

Egger test: p=0.915


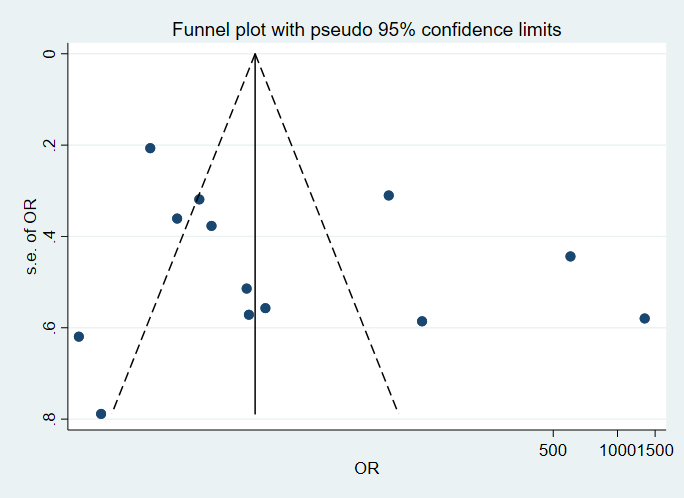


**Supplementary figure 1f: Funnel plot and Egger test for prevalence of microbleeds (Figure 6)**

Egger test: p=0.090


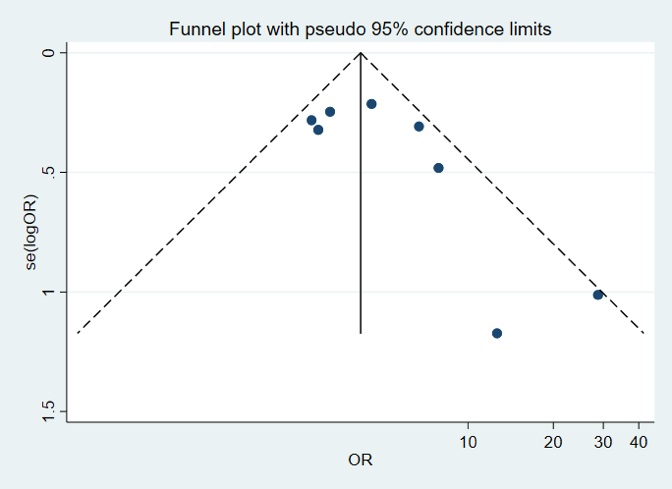

Supplement: euac003_Supplementary_Data [file euac003_supplementary_data.docx]
